# Application of Ionic Liquids in Hydrometallurgy

**DOI:** 10.3390/ijms150915320

**Published:** 2014-08-29

**Authors:** Jesik Park, Yeojin Jung, Priyandi Kusumah, Jinyoung Lee, Kyungjung Kwon, Churl Kyoung Lee

**Affiliations:** 1 School of Advanced Materials & Systems Engineering, Kumoh National Institute of Technology, Gumi, Kyungbuk 203-701, Korea; E-Mail: parkjesik@gmail.com; 2 Department of Energy & Mineral Resources Engineering, Sejong University, Seoul 143-747, Korea; E-Mails: yeojin1117@hanmail.net (Y.J.); priyandi.pk@gmail.com (P.K.); 3 Metallurgy Research Team, Korea Institute of Geoscience and Mineral Resources, Daejeon 305-350, Korea; E-Mail: jinlee@kigam.re.kr

**Keywords:** ionic liquid, hydrometallurgy, extraction, synthesis, processing, electrolysis

## Abstract

Ionic liquids, low temperature molten salts, have various advantages manifesting themselves as durable and environmentally friendly solvents. Their application is expanding into various fields including hydrometallurgy due to their unique properties such as non-volatility, inflammability, low toxicity, good ionic conductivity, and wide electrochemical potential window. This paper reviews previous literatures and our recent results adopting ionic liquids in extraction, synthesis and processing of metals with an emphasis on the electrolysis of active/light, rare earth, and platinum group metals. Because the research and development of ionic liquids in this area are still emerging, various, more fundamental approaches are expected to popularize ionic liquids in the metal manufacturing industry.

## 1. Introduction

There has been rapid development in electronic, informative, automotive and petrochemical industries in recent decades, and accordingly, the depletion of underground metal resources has become more and more serious. Solutions to the metal resources depletion could be the efficient extraction of low grade ores, and the recovery of valuable metals from urban mining. In addition, new environmentally friendly and less energy consumptive methods should be developed to relieve environmental and energy problems of the existing metal extraction technology.

In general, the whole metal extraction processes can be divided into: (1) pretreatment process; (2) extraction process; and (3) refining process. The pretreatment process is basically the mineral processing stage including crushing, grinding, screening, separation and so on. The extraction process can be classified as pyrometallurgy, reducing metal concentrates at high temperatures, and as hydrometallurgy, which is the process of recovering metals from solution media through dissolution, separation and purification steps. The refining process, which is also based on pyrometallurgy and hydrometallurgy principles, is usually required for increasing the purity of final metal products.

In the case of the steel making industry based on pyrometallurgy, efforts to reduce environmental issues such as exhaust gas, dust, slag, and waste water arising from the smelting process and to recover waste energy in the smelting process need to be expanded, in addition to the development of highly efficient smelting techniques for the treatment of low grade ores. Copper extractive metallurgy using high temperature smelting and electrolytic refining undergoes similar technological, environmental, and energy challenges to the steel making industry. Zinc manufacturing technology based on hydrometallurgy needs to consider an environmental issue of waste acid as well as the aforementioned pursuit of highly efficient and energy saving extractive methods. The development of energy saving techniques is critical, in particular, for aluminum making industry considering the current extractive technology based on high temperature molten salt electrolysis.

**Figure 1:**
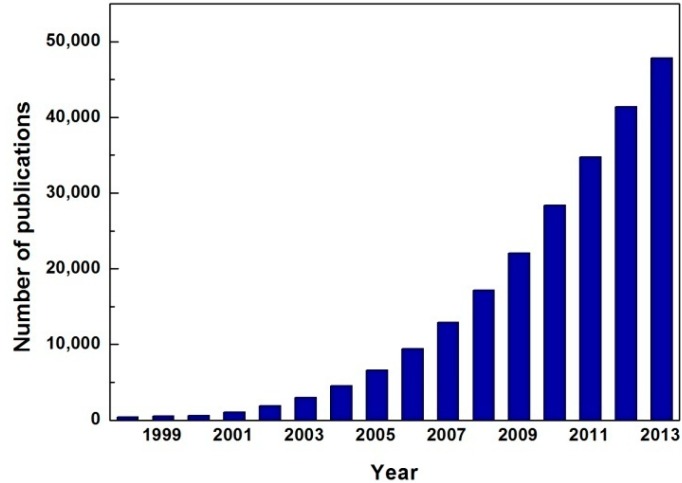
Annual growth of publications related to ionic liquids [2].

Among a variety of efforts to overcome the disadvantages of the existing metal manufacturing technology, non-aqueous solvents can be an alternative to the current acid media in hydrometallurgy. Ionic liquids, salts in the liquid state, are especially emerging in an effort to replace conventional water or organic solvents. Ionic liquids started to gain attention from the ethylammonium nitrate, a room temperature molten salt, synthesized in 1914 by Paul Walden, and relevant studies have exploded since 2000 as seen in Figure 1 where the number of publications related to ionic liquids is expressed from 1998–2013. The initial application of ionic liquids was restricted to media for polymer synthesis, but the application has expanded to various fields such reaction catalyst, electrolyte of energy storage devices, biosensor, separation/extraction agent, lubricant and so on [1]. Ionic liquids have a variety of advantages manifesting themselves as durable and environmentally friendly solvents—so called, green solvents. They have low vapor pressure, a non-flammable nature, and excellent chemical/electrochemical/thermal stability, which are favorable to long-term operation. More often than not, the term of task specific ionic liquids is used to emphasize their versatility in performing a specific task by designing their structure with countless combinations of cations and anions. Thus, the development of task specific ionic liquids for various kinds of industry including metal manufacturing is actively continued. This paper reviews previous literatures adopting ionic liquids in extraction, synthesis and processing of metals in the field of hydrometallurgy.

## 2. Results and Discussion

### 2.1. Ionic Liquids

**Figure 2:**
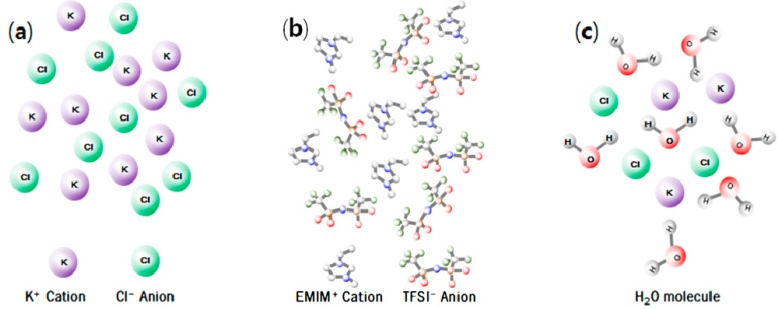
Schematic diagram of (**a**) molten KCl; (**b**) ionic liquid; and (**c**) aqueous KCl structures.

Ionic liquids are basically a family of molten salts comprising organic cations and organic/inorganic anions [3]. While common ionic salts at room temperature exist as a solid where cations and anions are alternately packed, ionic liquids have a lower melting point below 100 °C because cations and anions are not packed due to their large difference in ionic size and the resulting low lattice energy [4]. In particular, ionic liquids that exist as a liquid at room temperature are called room temperature ionic liquids (RTILs), and the number of currently known RTILs reaches about 10^6^ [5,6]. Figure 2 illustrates the structures of representative molten salt, ionic liquid, and aqueous solution.

A large percentage of ionic liquids have organic cations such as imidazolium or pyridinium, alkyl-substituted heterocyclic ring molecules, capable of ionic bonding with various kinds of anions. By a proper combination of cation and anion, we could control the melting point, density, viscosity, hydrophilicity/hydrophobicity of ionic liquids. Table 1 summarizes the unique properties of ionic liquids.

**Table 1:** Unique properties of ionic liquids.

Properties	Values
Melting point	Preferably below 100 °C
Liquidus range	Often > 200 °C
Thermal stability	Usually high
Viscosity	Normally < 100 cP, workable
Dielectric constant	Implied < 30
Polarity	Moderate
Ionic conductivity	Usually < 10 mS/cm
Molar conductivity	<10 Scm^2^/mol
Electrochemical window	Often > 4 V
Vapor pressure	Usually negligible

Contrary to the usual electrolyte where ionic salts are dissolved in a solvent, ionic liquids can act as electrolyte without the addition of solvent. Further, ionic liquids have very low vapor pressure suffering a negligible loss at room temperature and can even be vacuum dried [7,8]. Electrochemical stability and ionic conductivity are also very important properties of electrolyte to be considered as well as low vapor pressure. A large number of ionic liquids have wider electrochemical windows than 4 V, and some can have as wide as 6 V. Moreover, ionic liquids have a generally satisfactory ionic conductivity about 10 mS/cm, which can be comparable to that of sea water. However, they have a much larger viscosity than common organic solvents or water, which incurs a reduced ionic conductivity [9]. Thus, a combination of low viscosity and high ionic conductivity would be favorable to the usage of ionic liquids as electrolyte. The aforementioned features in addition to non-flammability make ionic liquids attractive to various applications such as organic synthesis [10,11], fine chemical production [12,13], and electrolytes for capacitors [14], dye-sensitized solar cells [15] and batteries [16].

**Figure 3:**
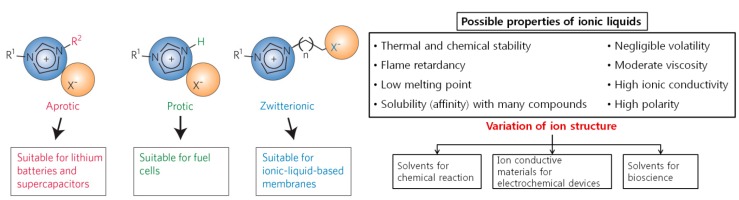
Design of ionic liquids for special purposes (Reprinted with permission from ref. [17] Copyright 2009 Rights Managed by Nature Publishing Group).

Favorable properties of ionic liquids are different depending on their applications, and the control of their properties is possible by a proper combination of cation and anion. For example, ionic liquids should be aprotic with low volatility, non-flammability, high ionic conductivity, and a wide potential window to be used as electrolytes for energy storage devices such as lithium batteries. On the other hand, ionic liquids need to be protic to be used as electrolytes for fuel cells while zwitterionic ionic liquids are preferable as membranes for metal extraction as illustrated in Figure 3.

**Figure 4:**
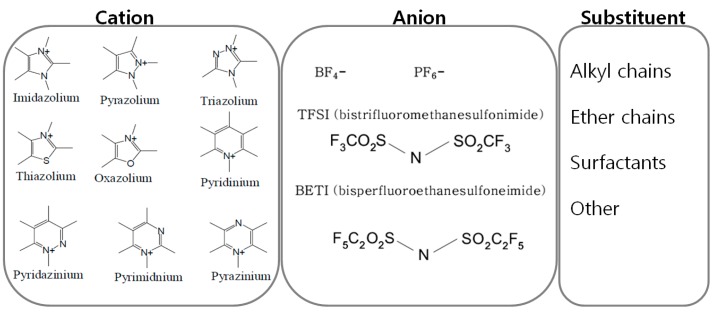
Species that are usually considered as cations, anions and substituents for designing task specific ionic liquids.

**Figure 5:**
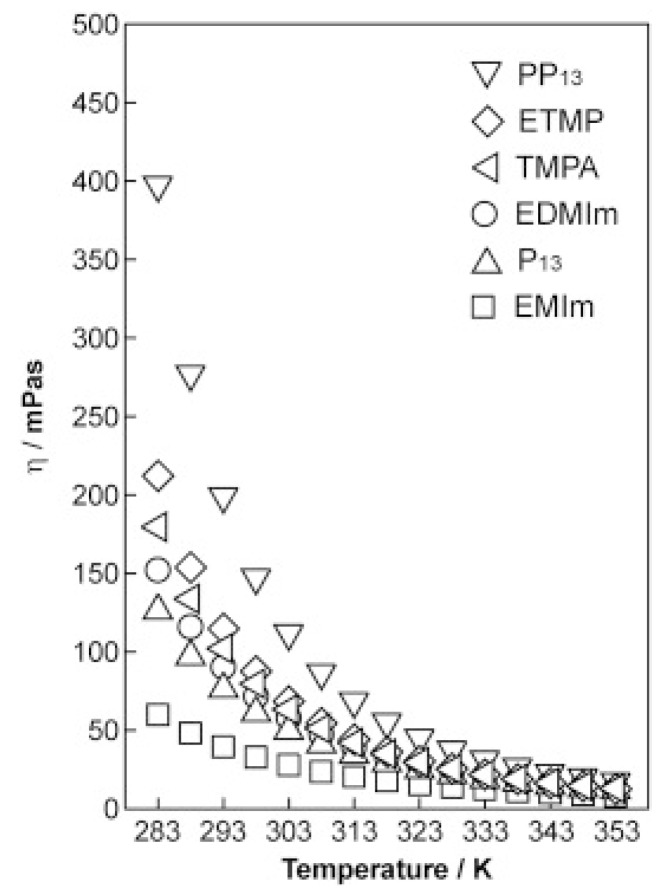
Effect of cations on the viscosity of ionic liquids (Reprinted with permission from ref. [18] Copyright 2010 Elsevier B.V).

Physicochemical properties of ionic liquids are basically determined by their components, that is, combinations of cations and anions. By combining cations and anions of different structure and size, the melting point, density, viscosity, and chemical reactivity can be controlled. The species in Figure 4 are usually considered in tuning the relevant properties of ionic liquids for cations, anions and substituents. For example, Figure 5 shows the temperature dependence of viscosity (η) for the six N(SO_2_CF_3_)_2_-anion-based RTILs. The obtained viscosities increase in the order EMIm (1-ethyl-3-methylimidazolium) < P13 (*N*-methyl-*N*-propylpyrrolidinium) < EDMIm (1-ethyl-2,3-dimethylimidazolium) < TMPA (trimethylpropylammonium) < ETMP (1-ethyl-2,3,5-trimethylpyrazolium) < PP13 (*N*-methyl-*N*-propylpiperidinium) at all measured temperatures.

**Figure 6:**
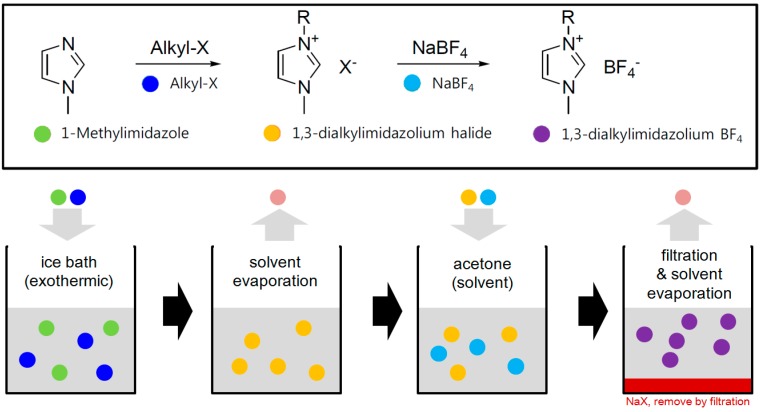
Synthesis procedure of an ionic liquid ([Dmim]BF_4_).

Although an ionic liquid could be synthesized by various routes, [Dmim]BF_4_, an imidazolium-based ionic liquid, can be prepared in a relatively simple method as illustrated in Figure 6. The synthesis steps are sequentially conducted as follows: quaternization of amine, solvent removal, anion exchange with a metal salt, solvent/salt removal and refining. Using this method, a wide spectrum of physicochemical property of ionic liquids can be obtained by different functionalization (through the variation of alkyl-X) and anion species (through the variation of metal salt).

**Figure 7:**
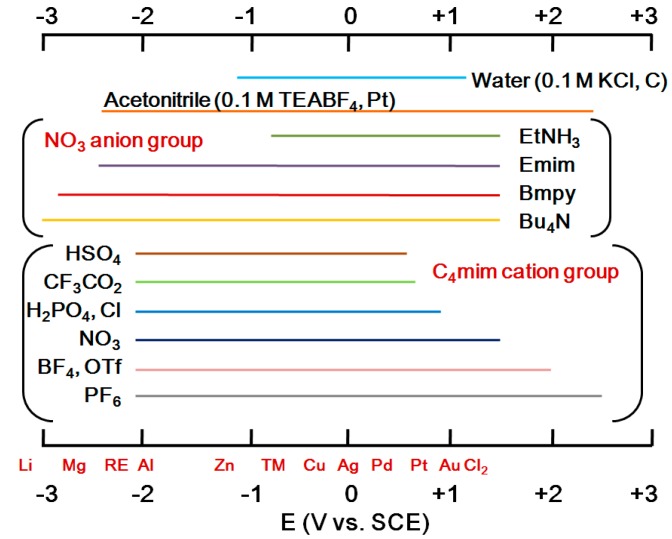
Effect of ion group on electrochemical window of ionic liquids.

Electrochemical characteristics such as electrochemical window and ionic conductivity can be controlled as well. Figure 7 shows the dependency of electrochemical window of ionic liquids on their constituting cations and anions. The stability limits of electrochemical window with respect to oxidation and reduction are respectively determined by anion and cation of an ionic liquid. Meanwhile, the ionic conductivity of ionic liquids can be regulated indirectly by adjusting the viscosity of ionic liquids and taking the inverse relationship between conductivity and viscosity into account. The viscosity and the according ionic conductivity are usually varied by changing the type and size of functional groups of cations or by selecting different kinds of anions. For instance, in the choice of anions, relatively small anions such as BF_4_ and PF_6_ known to have high conductivity were often considered as anions of ionic liquids, whereas TFSI anion is nowadays most frequently adopted thanks to its improved stability.

**Figure 8:**
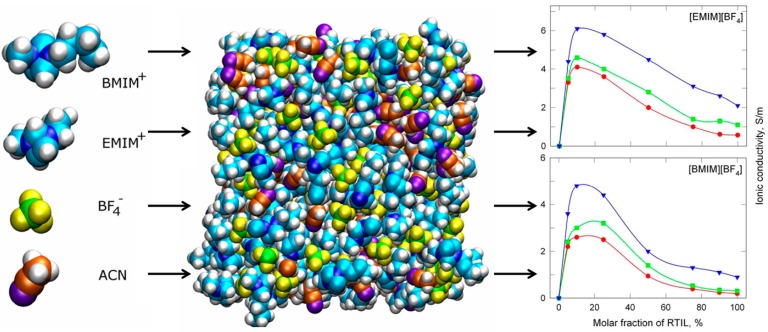
Enhancement of ionic conductivity of ionic liquids by mixing with organic solvents (Reprinted with permission from ref. [19] Copyright 2012 American Chemical Society).

**Figure 9:**
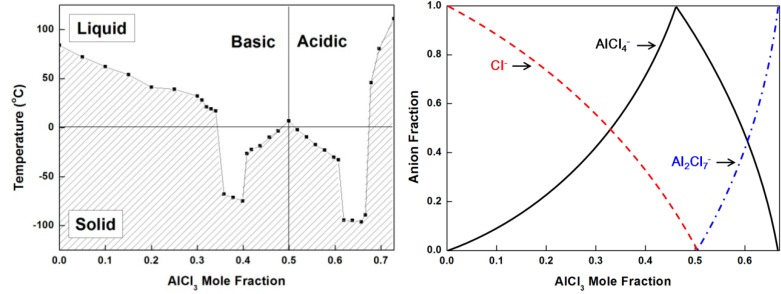
Phase diagram and complexation behavior of [C_2_C_1_im]Cl-AlCl_3_ (Reprinted with permission from ref. [20,21] Copyright 1984 American Chemical Society).

To overcome the intrinsic limitation of properties belonging to ionic liquids, conventional solvents could be incorporated. Chaban *et al.* reported that the addition of acetonitrile to BF_4_ anion-based ionic liquid can improve the ionic conductivity in Figure 8 [19]. Meanwhile, the understanding of physicochemical properties of metal salt-containing ionic liquids is also important for the application of these materials to hydrometallurgy or electrometallurgy. Figure 9 is an example to investigate the phase diagram and complexation behavior of Al salt-containing ionic liquids.

### 2.2. Extraction of Metals

**Figure 10:**
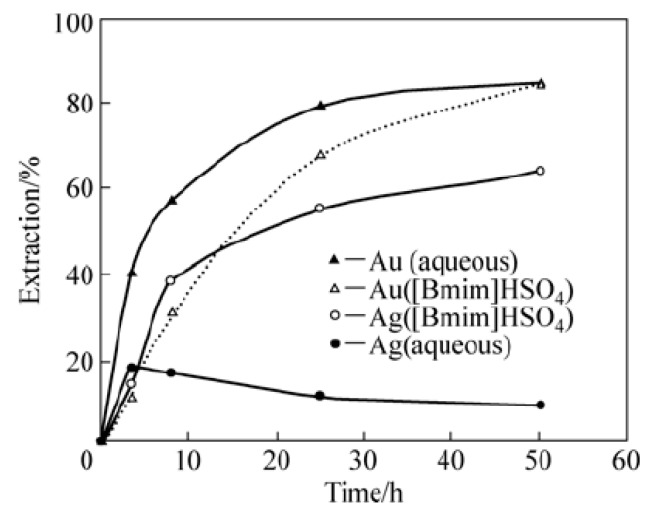
Leaching efficiency of Au/Ag using aqueous sulfuric acid or [Bmim]HSO_4_ with Fe_2_(SO_4_)_3_ (Reprinted with permission from ref. [24] Copyright 2010 Elsevier B.V).

Ionic liquids have been introduced to leaching and solvent extraction processes in the extraction of various metals. For example, ionic liquids can be an alternative leaching agent for copper ore to the existing leaching techniques based on hydrochloric acid leaching agent, high temperature/pressure operation or bioleaching. 1-butyl-3-methylimidazolium tetrafluoroborate ([Bmim]BF_4_) ionic liquid as a solvent and Fe(BF_4_)_3_ as an oxidizer were used for chalcopyrite leaching, and 90% of copper was gained at 100 °C after 8 h leaching by McCluscey *et al.* [22]. Whitehead *et al.* [23] used [Bmim]HSO_4_ ionic liquid with Fe_2_(SO_4_)_3_ in the leaching of gold and silver, which demonstrates the potential of ionic liquids as a selective leaching agent for precious metals with an advantage of replacing toxic acid agents as shown in Figure 10.

**Figure 11:**
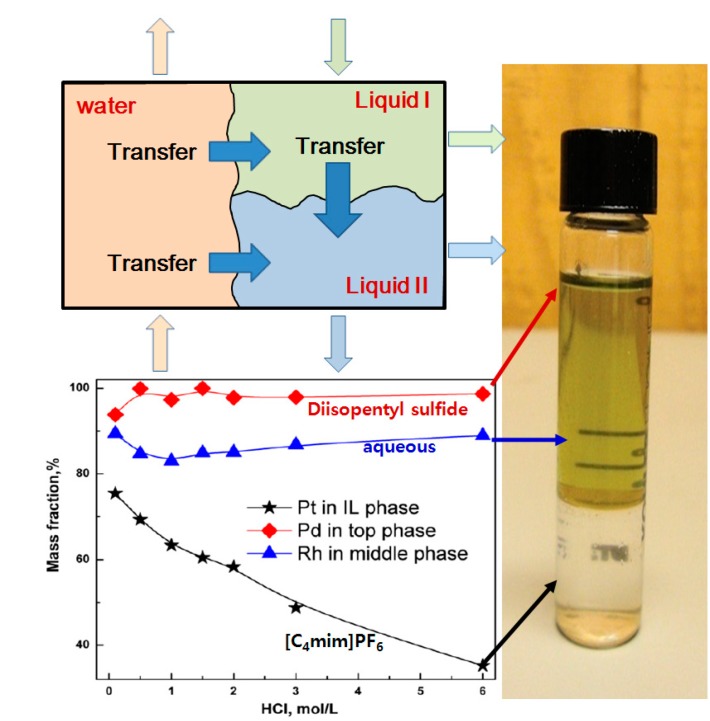
Extraction behaviors of Pt(IV), Pd(II) and Rh(III) in three-liquid-phase system (Reprinted with permission from ref. [25] Copyright 2013 Elsevier B.V).

Zhang *et al.* [25] suggested a solvent extraction technique based on three-liquid-phase partitioning including ionic liquid phase, which enables the separation or concentration of Pt(IV), Pd(II), Rh(III) from hydrochloric acid leaching solution by using [C_4_mim]PF_6_–oil phase (diisopentyl sulfide-nonane organic solution)–water phase (hydrochloric acid aqueous solution). While Rh(III) remained in the middle water phase, Pd(II) and Pt(IV) migrated toward the upper oil phase and the lower ionic liquid phase respectively as shown in Figure 11. Their application of ionic liquid to the solvent extraction of platinum group metals illustrates the effectiveness of ionic liquid in the selective separation from multi-metal solutions compared to the conventional two-phase solvent extraction systems. Cieszynska *et al.* [26] reported that Pd(II) can be extracted selectively from hydrochloric acid solutions containing multi-metal ions by using extraction agents (CyphosIL 101 and CyphosIL 104) based on ionic liquids (trihexyl(tetradecyl)phosphonium chloride and trihexyl(tetradecyl)phosphoniumbis-2,4,4-trimethylpentylphosphinate), respectively. They mixed the extraction agents with toluene and were able to extract more than 99% Pd(II) from 0.1 M hydrochloric acid solution including Pd(II), Ni(II), Cu(II), Pb(II), Fe(III), Rh(III), Ru(III), and Pt(IV).

These research results suggest the favorable application of ionic liquids to the separation and recovery of platinum group metals with considering the fact that the recovery in the conventional aqueous system is complex and difficult. Moreover, ionic liquids can be directly used as electrolyte for the electrowinning of dissolved metal ions because they have high ionic conductivity and wide potential windows enabling the electrolytic reduction of various metals. Meanwhile, Tian *et al.* [24] and Binnemans *et al.* [27,28,29,30,31,32,33,34,35,36] focused on the usage of ionic liquids in the extraction of nonferrous metals, which broadened the application of ionic liquids from precious metals to light and rare earth metals. In particular, in the separation between rare earth metals and transition metals, Binnemans *et al.* have investigated many combinations of metals and ionic liquids in the liquid-liquid extraction so far.

**Figure 12:**
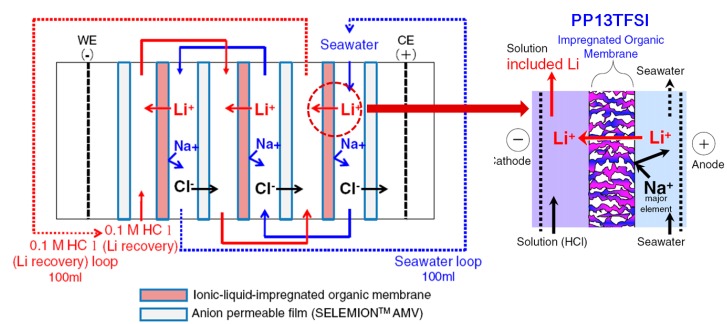
Schematic of the SILMs; Li extraction from sea water (Reprinted with permission from ref. [38] Copyright 2013 Elsevier B.V).

Ionic liquids can also be used in metallurgy in a form of supported ionic liquid membranes (SILMs), which stabilize ionic liquids by using porous supporters. The disadvantageous high viscosity of ionic liquid compared to organic solvents can be mitigated by using SILMs, leading to the application of CO_2_ separation or metal ion-exchange membranes [37]. Hoshino [38] suggested the application of SILMs to the recovery of lithium from seawater as shown in Figure 12. The SILMs can recover valuable metals from solutions where target metals are dissolved by selecting an adequate ionic liquid, which have superior solubility of target metals and are immiscible with aqueous solutions, such as *N*-methyl-*N*-propylpiperidinium bis(trifluoromethanesulfonyl)imide (PP13TFSI) for lithium recovery.

### 2.3. Electrolysis of Metals

Ionic liquids have been adopted more extensively on the field of metal electrolysis than the other fields belonging to metal manufacturing industry. Because there are too many references in this field to cover their details in this review, we try to restrict the contents of this section to our own results. Table 2 is the literature survey result where the combination of some selected metals and ionic liquids is specified with reference information. Readers may refer to other review papers including F. Endre’s [39,40,41] for a more extensive overview in this field.

Table 2Literature survey result for the combination of some selected metals and ionic liquids in the field of metal electrolysis (**A**–**G**).

**Table:** (**A**)

	Metal	Li	Mg	Au	Pt	Pd
Ionic Liquids						
BMI[TFSI]				[42]		
BMP[TFSI]					[43]	[44]
Bu_3_HexP^+^[TFSI]^−^		[45]				
Bu_3_HexN^+^[TFSI]^−^		[45]				
C_3_mpyr[TFSI]		[46]				
DEME[TFSI]			[47,48]			
EMI[TFSI]		[49]				
EMIm[TFSI]		[50]				
N_1113_[TFSI]		[49]				
N_1114_[TFSI]		[51]

**Table:** (**B**)

	Metal	Li	Mg	Au	Ta	Si	Nd
Ionic Liquids							
PP13[TFSI]			[52]				
Pyr14[TFSI]						[53]	
TMHA[TFSI]						[54,55]	
C_3_mpyr[FSI]		[56]					
BMP[TFSA]				[57]			
EMIm[TFSA]				[58]			
PMIm[TFSA]						[59]	
PP13[TFSA]		[60,61]					
P_2225_[TFSA]							[62]
Py14[TFSA]		[60,63]			[64]

**Table:** (**C**)

	Metal	Li	Mg	Au	Pt	Ti	Ta	Si	In
Ionic Liquids									
TMHA[TFSA]		[60]							
EMI[FSA]		[61]							
EMI[TSAC]		[49]							
N_2222_[TSAC]		[49]							
ABN1*n*[Tf_2_N]		[65]							
BEPip[Tf_2_N]									[66]
BMIm[Tf_2_N]						[67]			
BMP[Tf_2_N]			[68]			[69]	[67,70,71,72,73]	[67,69,74,75]	[76]
C_3_(OH)_2_mim[Tf_2_N]					[77]				
C_4_Mim[Tf_2_N]				[78]	[78]

**Table:** (**D**)

	Metal	Mg	Au	Pt	Pd	Ti	Si
Ionic Liquids							
C_10_MIm[Tf_2_N]			[79]				
EMIm[Tf_2_N]						[80]	
P_66614_[Tf_2_N]			[81]	[81,82]			
P(C_6_)_3_C_14_[Tf_2_N]			[83]	[83,84,85]			
Py14[Tf_2_N]							[86,87,88]
TBMA[Tf_2_N]			[89]				
BMP[TfO]		[68]					
BMIm[Cl]			[90]	[90]	[91,92,93,94,95,96,97,98]	[99,100]	
C_3_COOH[Cl]				[77]			
C_3_OHMIm[Cl]				[77]

**Table:** (**E**)

	Metal	Li	Au	Pt	Ti
Ionic Liquids					
C_3_CNmim[Cl]				[77]	
C_3_(OH)_2_mim[Cl]				[77]	
C_3_(OCOCH_2_SH)_2_mim				[77]	
BMECl[AlCl_3_]		[101]			
BMIC[AlCl_3_]					[102]
BTMAC[AlCl_3_]				[103]	
EMICl[AlCl_3_]		[51,104]			
EMImCl[AlCl_3_]		[105]			
EtMelmCl[AlCl_3_]					[106,107]
BMI[BF_4_]			[108]

**Table:** (**F**)

	Metal	Mg	Au	Pt	Pd	Ti	In
Ionic Liquids							
BMIm[BF_4_]		[52,109,110,111,112]		[113]			
BMMIm[BF_4_]						[114]	
C_3_CNMIm[BF_4_]				[77]			
C_3_(OH)_2_mim[BF_4_]				[77]			
DEME[BF_4_]				[115]			
EMI[BF_4_]			[108]				
EMIm[BF_4_]		[68]	[67]		[67]		[67]
BMIm[BR]			[116]				
BMIm[BTA]						[117,118]	
BMP[DCA]			[119]	[43]	[120,121]

**Table:** (**G**)

	Metal	Au	Pt	Pd	In
Ionic Liquids					
BMIm[PF_6_]		[122]	[113,123]		
C_3_(OH)_2_mim[PF_6_]			[77]		
C8Py[PF_6_]				[124]	
ZnCl_2_[EMIC]			[125,126,127]		
ZnCl_2_[EMIm-Cl]			[67]		
EMI[Cl-BF_4_]		[42]		[42]	[128]
mercapto IL[1-methyl-3(2'-mercaptoacetoxyethyl) imidazoliumhexafluorophosphate]		[129]	[129]	[129]	
TOMAC(Aliquat 336 chloride-IL)				[98,130]	
TOMAN(Aliquat 336 nitrate-IL)				[130]	

Active/light metals such as magnesium, rare earth metals such as neodymium and silicon are usually recovered by pyrometallurgical processes where metal phases are obtained by direct melting or by reduction with reductants often after the additional formation steps of specific metal salts. Generally these recovery processes have problems such as high operating temperature (600–2000 °C), high operating cost, low energy efficiency (50%–80%), high energy consumption (~100 kWh/kg metal), environmental issues, and waste management. Alternatively, these metals could be recovered by electrolysis, but its practicability is limited by the use of conventional aqueous electrolytes because of low reduction potential and high chemical activity of the metals in aqueous systems. However, the properties of ionic liquids such as wide electrochemical window, low vapor pressure and satisfactory ionic conductivity shed light on the expansion of electrolysis to the recovery of active/light metals, rare earth metals and silicon.

**Figure 13:**
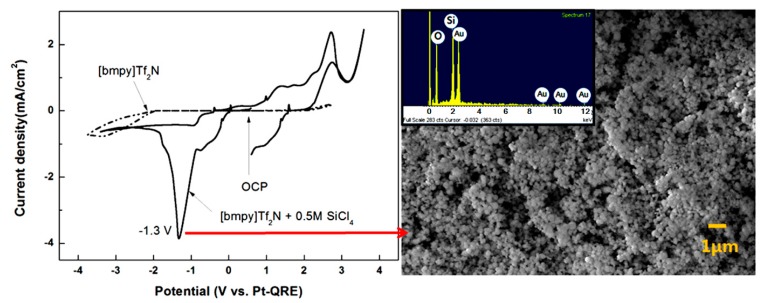
Electrochemical behavior of silicon in [Bmpy]Tf_2_N and electrodeposited silicon thin film [131].

For example, the electrochemical reduction behavior of silicon at room temperature was examined by using two kinds of ionic liquids (1-butyl-3-methylpyridinium bis(trifluoromethylsulfonyl)imide ([Bmpy]Tf_2_N) and 1-ethyl-3-methylimidazolium bis(trifluoromethylsulfonyl)imide ([Emim]Tf_2_N)) of which cations were different, and the resulting electrodeposited silicon thin films were obtained with the added salt of SiCl_4_ as shown in Figure 13 [131]. Further, the recycling of silicon single crystal cutting sludge was suggested based on the above reduction behavior of silicon in the ionic liquids, emphasizing that the proper choice of cation in ionic liquids is very important for the effective silicon recycling [132].

**Figure 14:**
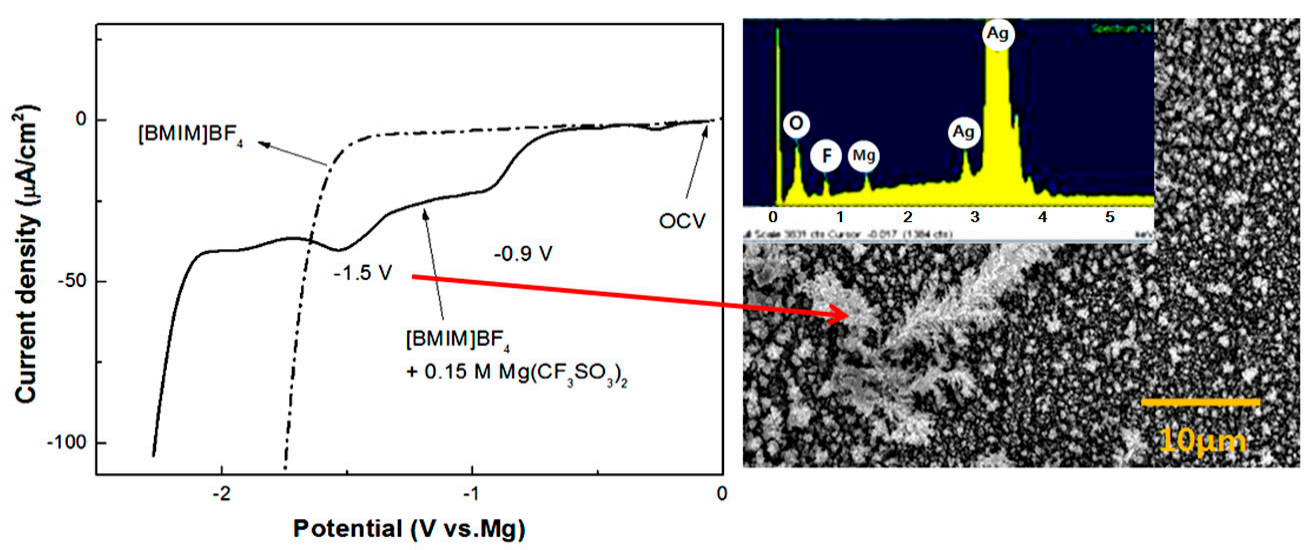
Electrochemical behavior of magnesium in [Bmim]BF_4_ and its electrodeposited thin film [133].

The electrowinning of magnesium, a representative light metal, is not feasible in aqueous electrolytes at room temperature because of its low reduction potential and high reactivity with water. However, the electrodeposition of magnesium could be possible when an electrochemically stable ionic liquid is used as electrolyte. This possibility was examined by adopting an imidazolium-based ionic liquid and Mg(CF_3_SO_3_)_2_ as a magnesium source [133]. The magnesium electrodeposited at a potential of 1.5 V (*vs.* Mg) has a particulate morphology as depicted in Figure 14. Although the electrodeposits on silver substrate include some impurities of F and O, which might originate from the Mg salt, in addition to Mg, this study showed that a careful choice of ionic liquid and metal salt enables the room temperature electrodeposition of active/light metals.

**Figure 15:**
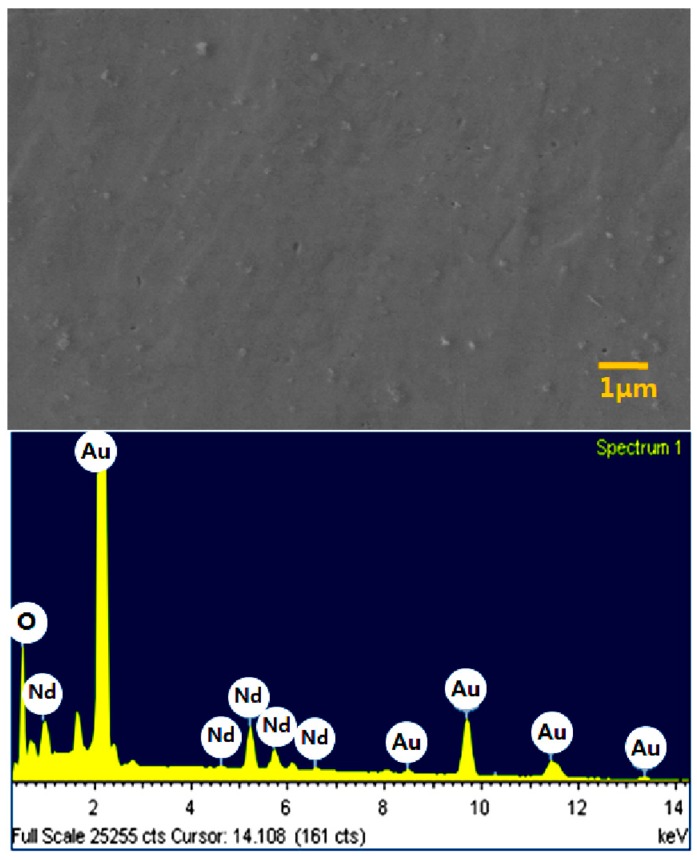
Electrodeposition of neodymium in [Emim]TFSI.

Rare earth metals are extensively used in high-tech industries despite their scarcity on earth. For example, neodymium, of which usage has been expanded in recent years because of a proliferation of high-performance magnets for advanced motors in electric vehicles, needs to be reused or recycled considering its limited availability. The conventional extraction method of neodymium metal is fused salt electrolysis, which has some disadvantages such as high operating cost, high energy consumption, environmental issues of chlorine gas emission and so on. The production of neodymium compounds also needs complicated and costly separation and purification stages. By contrast, Figure 15 shows that simple and relatively low-cost production and recycling of neodymium metal are possible by electrowinning or electrorefining of waste magnets in ionic liquids. This possibility was examined by using a gold substrate and 1-ethyl-3-methylimidazolium bis(trifluoromethylsulfonyl)imide ([Emim]TFSI) containing Nd(TFSI)_3_ as a neodymium source.

**Figure 16:**
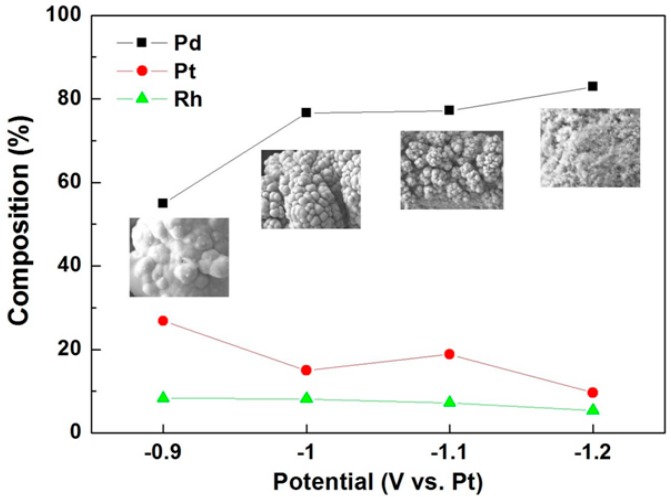
Compositional change of platinum group metals in [Bmim]Cl depending on applied potentials.

For the selective separation/recovery of platinum group metals, hydrometallurgy or pyrometallurgy techniques could be used. A leaching process based on hydrometallurgy adopts strong acid leaching agents, which can dissolve and recover various kinds of platinum group metals constituting automotive catalysts, however, in large quantities and which incurs environmental problems [134]. On the other hand, pyrometallurgical techniques need substantial energy input and large-scale facilities because crushing and grinding pre-treatment and high temperature smelting of platinum group metal-containing materials such as spent catalysts are required in these techniques. To overcome the disadvantages of the established techniques based on hydrometallurgy or pyrometallurgy, a newly proposed separation/recovery of platinum group metals by using ionic liquids is investigated as seen in Figure 16 where platinum, palladium and rhodium have different reduction behavior depending on applied potential.

## 3. Conclusions

Ionic liquids are a family of molten salts, comprising cations and anions, which exist as a liquid phase at low temperatures usually below 100 °C. Ionic liquids are considered to be durable and environmentally friendly solvents because they have low vapor pressure, a non-flammable nature, and excellent chemical/electrochemical/thermal stability, which are favorable to long-term operation. Further, they are versatile in performing a specific task by designing their structure with countless combinations of cations and anions. Thus, the development of task specific ionic liquids for the extraction, synthesis and processing of metals is actively aimed for in the metal manufacturing industry.

Ionic liquids have been introduced to leaching and solvent extraction processes in the extraction of various metals, and they can be directly used as electrolyte for the electrowinning of dissolved metal ions because they have high ionic conductivity and wide potential windows enabling the electrolytic reduction of various metals. Physicochemical and electrochemical properties of ionic liquids such as melting point, density, viscosity, chemical reactivity, ionic conductivity and electrochemical window can be optimized in principle for specific tasks by the proper design of cations and anions. Incidentally, the mixing of ionic liquids with conventional solvents has been attempted in order to widen the intrinsic limitations of properties belonging to ionic liquids.

Although there have been a number of papers regarding ionic liquids so far, relevant research activities in the field of hydrometallurgy have had limited success. Because the research and development of ionic liquids in this area are still emerging, various, more fundamental approaches should be helpful to popularize ionic liquids in the metal manufacturing industry.
